# Surgical outcomes of thoracoscopic extended thymectomy via subxyphoid and left intercostal approach

**DOI:** 10.3389/fsurg.2026.1795630

**Published:** 2026-03-25

**Authors:** Zhenlin Jiang, Zuxin Dong, Junting Chen, Jianhua Yan

**Affiliations:** Department of Thoracic Surgery, Hunan Provincial People’s Hospital (The First Affiliated Hospital of Hunan Normal University), Changsha City, Hunan, China

**Keywords:** intercostal approach, postoperative pain, subxiphoid approach, thoracoscopic surgery, thymectomy

## Abstract

**Background:**

Minimally invasive thymectomy is widely adopted for the management of thymic diseases and myasthenia gravis (MG). Among thoracoscopic techniques, the intercostal approach (ICA) is the most commonly used, whereas the subxiphoid approach (SA) has been proposed to reduce postoperative pain. However, comparative evidence remains heterogeneous.

**Methods:**

This single-center retrospective cohort study included consecutive patients who underwent minimally invasive thymectomy between January 2019 and December 2023. Patients were assigned to the ICA or SA group according to the surgical approach performed. The primary outcome was early postoperative pain assessed using the visual analog scale (VAS) within 48 h. Secondary outcomes included operative and perioperative parameters, postoperative complications, and short-term follow-up outcomes. Multivariable linear regression analysis was conducted to adjust for potential confounders.

**Results:**

Of 168 patients assessed for eligibility, 90 were included in the final analysis (ICA, *n* = 48; SA, *n* = 42). Baseline characteristics were comparable between groups. Operative time, intraoperative blood loss, duration of chest drainage, length of hospital stay, and hospitalization costs did not differ significantly. Early postoperative pain was lower in the SA group, with significantly reduced VAS scores during the first 48 h. Postoperative complication rates were low and similar between approaches, with no perioperative mortality observed. Among patients with MG, short-term neurological improvement did not differ significantly. Multivariable analysis confirmed that the subxiphoid approach was independently associated with lower early postoperative pain.

**Conclusions:**

Intercostal and subxiphoid thoracoscopic thymectomy demonstrated comparable perioperative safety and short-term clinical outcomes in this cohort. The subxiphoid approach was associated with lower early postoperative pain, while other operative and recovery-related parameters were similar. These findings support individualized surgical decision-making rather than a universally superior approach.

## 1 Introduction

Thymectomy is a cornerstone in the management of thymic epithelial tumors and has a well-established therapeutic role in patients with myasthenia gravis (MG), particularly in those with generalized disease or thymoma-associated MG (1–3). The evolution of surgical techniques over the past two decades has led to a paradigm shift from open median sternotomy toward minimally invasive approaches, driven by the desire to reduce surgical trauma while preserving oncological and neurological efficacy (4, 5). Video-assisted thoracoscopic surgery (VATS) thymectomy has therefore become widely adopted and is now considered a standard option in appropriately selected patients (6). Among minimally invasive techniques, the intercostal thoracoscopic approach (ICA) has been the most commonly utilized due to its technical familiarity, reproducibility, and direct access to the anterior mediastinum (7, 8). ICA allows adequate exposure for complete thymic resection and has demonstrated favorable perioperative outcomes and oncological safety in multiple series (9, 10). However, the requirement for intercostal incisions raises concerns regarding postoperative pain related to intercostal nerve irritation, which may negatively influence early recovery, mobilization, and patient-reported outcomes (11). To address these limitations, the subxiphoid approach (SA) has been introduced as an alternative minimally invasive route for thymectomy. By avoiding intercostal spaces and providing a midline caudal view of the anterior mediastinum, SA has been proposed to reduce postoperative pain and improve visualization of bilateral phrenic nerves, potentially facilitating extended thymectomy (7, 12, 13). Several reports have suggested that SA may be associated with lower postoperative pain scores, improved cosmetic results, and faster early recovery when compared with ICA (14–16). These potential advantages have generated increasing interest in the subxiphoid technique, particularly in high-volume thoracic centers. Nevertheless, SA is not without limitations. The technique is technically more demanding, often requires carbon dioxide insufflation, and may be associated with longer operative times, especially during the learning curve (16, 17). In addition, concerns have been raised regarding its applicability in patients with laterally located tumors, larger thymic lesions, or unfavorable cardiopulmonary anatomy, where exposure through a subxiphoid route may be restricted (18). Consequently, the choice between ICA and SA remains largely dependent on surgeon preference, institutional experience, and individual patient characteristics. Despite a growing number of comparative studies, the current literature remains inconclusive. Prior reports vary substantially in study design, patient selection criteria, outcome definitions, and statistical methodology (16, 19, 20). Many studies are limited by small sample sizes or lack appropriate adjustment for baseline differences, which may introduce selection bias, particularly in retrospective analyses. Furthermore, while postoperative pain is frequently cited as a potential advantage of SA, its role as a primary outcome has not been consistently emphasized, and results across studies remain heterogeneous. In this context, a clearer understanding of the relative benefits and trade-offs between ICA and SA in routine clinical practice is needed. The present single-center retrospective cohort study aims to compare perioperative and short-term outcomes of intercostal and subxiphoid thoracoscopic thymectomy, with a particular focus on postoperative pain. By systematically evaluating operative parameters and early clinical recovery, this study seeks to provide balanced evidence to support individualized surgical decision-making rather than advocating a universally superior approach.

## 2 Material and methods

### 2.1 Study design and ethical approval

This study was designed as a single-center retrospective cohort analysis conducted at a tertiary thoracic surgery referral center. Consecutive patients who underwent minimally invasive thymectomy between January 2019 and December 2023 were included. The study protocol was reviewed and approved by the institutional ethics committee, and the requirement for written informed consent was waived owing to the retrospective nature of the study and anonymized data analysis.

### 2.2 Patient selection

Patients were eligible for inclusion if they underwent thoracoscopic thymectomy for thymic epithelial tumors or non-thymomatous thymic lesions, with or without associated myasthenia gravis. Only minimally invasive procedures were considered. Exclusion criteria included conversion to open surgery, tumor size greater than 5 cm on preoperative imaging, radiological or intraoperative evidence of invasion into adjacent organs, history of ipsilateral thoracic surgery, or incomplete perioperative clinical data. Patients were allocated to one of two groups based on the surgical approach used: intercostal thoracoscopic approach (ICA) or subxiphoid approach (SA). The choice of approach was determined by tumor characteristics, anatomical considerations, and surgeon preference.

### 2.3 Preoperative evaluation and data collection

All patients underwent standard preoperative evaluation, including chest computed tomography to assess tumor size, location, and laterality. Demographic variables, comorbidities, myasthenia gravis status, and relevant clinical information were retrieved from electronic medical records. Histopathological diagnosis was obtained from postoperative pathology reports, and thymic tumors were classified according to established pathological criteria.

Preoperative characterization was based on contrast-enhanced chest CT, focusing on lesion size, laterality, morphology, margins, and radiologic signs suggestive of invasion. Preoperative biopsy was not routinely performed because all included lesions were deemed resectable on imaging and scheduled for therapeutic thymectomy; histopathological diagnosis was therefore established on the resected specimen. Lesions ultimately classified as non-thymomatous were identified by postoperative pathology (e.g., thymic cysts, hyperplasia, or other benign entities).

### 2.4 Surgical techniques

All procedures were performed under general anesthesia with single-lung ventilation. Operations were conducted by a dedicated thoracic surgery team consisting of three consultant thoracic surgeons. During the study period, the intercostal thoracoscopic approach (ICA) represented the established technique, whereas the subxiphoid approach (SA) was introduced following institutional training and adoption. Consequently, the choice of surgical approach reflected tumor characteristics, anatomical considerations, and surgeon preference, and a potential learning-curve or temporal (era) effect cannot be fully excluded. In the ICA group, thymectomy was performed via a left-sided thoracoscopic approach using three intercostal ports positioned along the lateral chest wall, including one camera port and two working ports, thereby providing direct access to the anterior mediastinum. In the SA group, a transverse subxiphoid incision was created inferior to the xiphoid process, with an additional auxiliary thoracoscopic port inserted as required for instrumentation. Carbon dioxide insufflation was selectively applied in both approaches to optimize mediastinal exposure. In all cases, the surgical objective was complete resection of the thymus gland together with surrounding mediastinal adipose tissue while carefully preserving the bilateral phrenic nerves. A chest drain was routinely placed at the conclusion of the procedure.

### 2.5 Postoperative management

Postoperative analgesia followed an institutional standardized regimen for both groups. Scheduled analgesics included intravenous flurbiprofen axetil (50 mg, every 12 h) beginning in the post-anesthesia care unit. Rescue analgesia was administered according to predefined criteria (VAS ≥ 4 or patient request) using intravenous sufentanil (5–10 μg per dose). Use of regional anesthesia techniques (e.g., erector spinae plane block, paravertebral block, intercostal nerve block) was not routinely performed to minimize confounding variables in the assessment of incision-related pain, and, when used, was recorded and compared between groups.

### 2.6 Outcome measures

The primary outcome of the study was postoperative pain intensity, assessed using VAS scores during the early postoperative period. Secondary outcomes included operative time, intraoperative blood loss, duration of chest drainage, length of postoperative hospital stay, postoperative complications, and short-term clinical outcomes. Postoperative complications were defined as any adverse events occurring during hospitalization or within 30 days after surgery.

### 2.7 Follow-up and assessment of clinical outcomes

Patients were routinely followed in the outpatient clinic after discharge. For patients with myasthenia gravis, short-term neurological outcomes were evaluated during follow-up visits and categorized according to clinical symptom improvement and disease control. Tumor recurrence was assessed using follow-up imaging when available.

### 2.8 Statistical analysis

Statistical analyses were performed using standard statistical software. Normality of continuous variables was assessed using the Shapiro–Wilk test. Normally distributed variables are presented as mean ± standard deviation (SD) and were compared using the Student's t-test, whereas non-normally distributed variables are presented as median with interquartile range (IQR) and were compared using the Mann–Whitney U test. Categorical variables are expressed as frequencies and percentages and were compared using the chi-square test or Fisher's exact test, as appropriate. To minimize the impact of baseline differences between groups, multivariable linear regression analysis was conducted adjusting for age, sex, tumor size, tumor laterality, and presence of myasthenia gravis. All statistical tests were two-sided, and a *P* value < 0.05 was considered statistically significant.

## 3 Results

### 3.1 Patient characteristics

**Figure 1:**
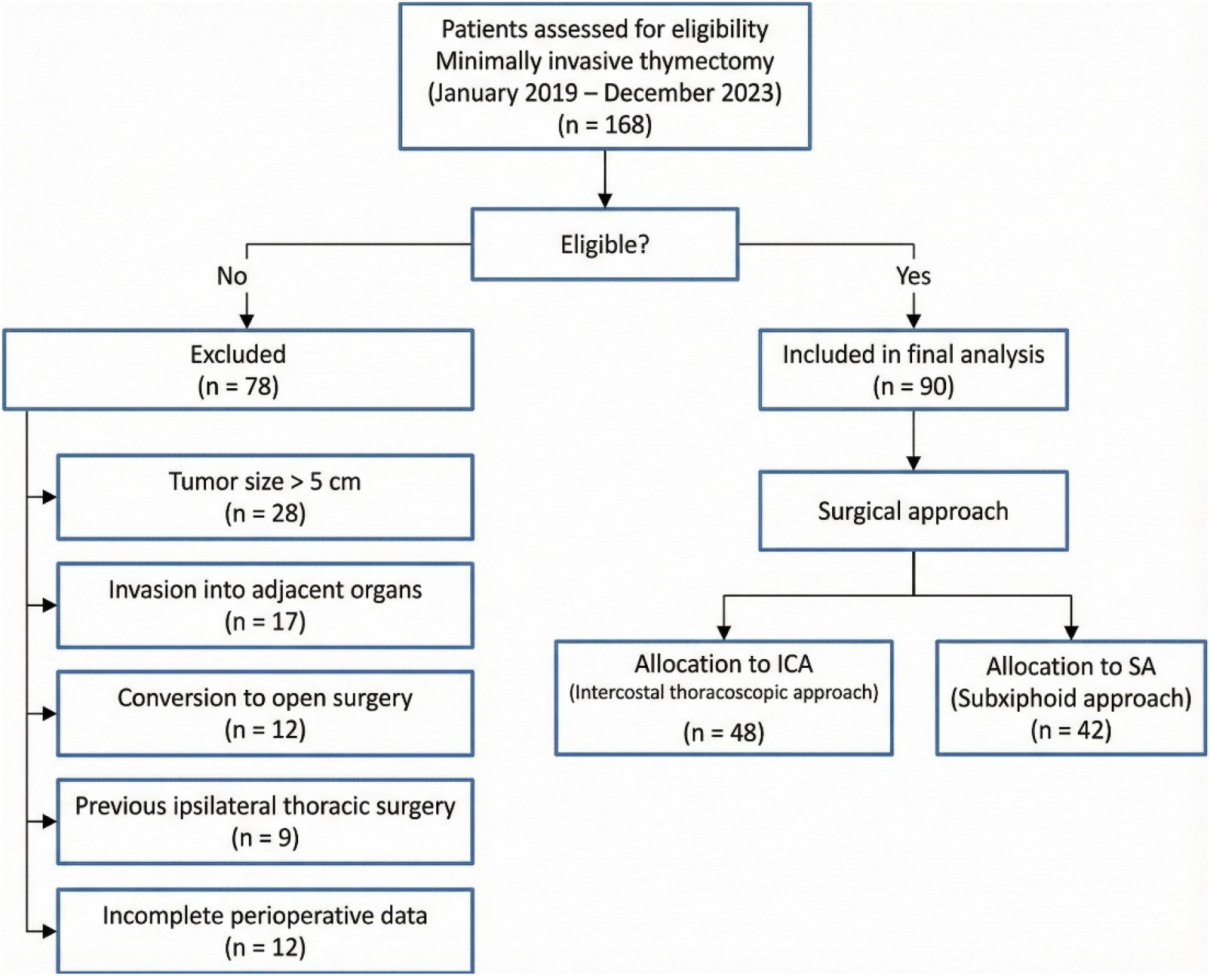
Study flow diagram and patient selection. Flow diagram illustrating patient selection and allocation in this retrospective cohort study. A total of 168 patients undergoing minimally invasive thymectomy between January 2019 and December 2023 were assessed for eligibility. After exclusion based on predefined criteria, 90 patients were included in the final analysis and allocated to the intercostal thoracoscopic approach (ICA) or the subxiphoid approach (SA) according to the surgical technique used.

During the study period, 168 patients underwent minimally invasive thymectomy, of whom 90 met the inclusion criteria and were included in the final analysis (ICA: *n* = 48; SA: *n* = 42) (Figure 1). Baseline characteristics were comparable between groups (Table 1). Mean age was 46.8 ± 12.3 years in the ICA group and 44.9 ± 11.7 years in the SA group (*P* = 0.42). Female patients accounted for 56.3% (27/48) in the ICA group and 54.8% (23/42) in the SA group (*P* = 0.88). Mean tumor size was 3.2 ± 0.9 cm in the ICA group and 3.1 ± 1.0 cm in the SA group (*P* = 0.67). Myasthenia gravis was present in 54.2% (26/48) and 59.5% (25/42) of patients in the ICA and SA groups, respectively (*P* = 0.61). The distribution of pathological diagnoses did not differ significantly (thymoma: 70.8% vs. 73.8%, *P* = 0.81).

**Table 1:** Baseline demographic and clinical characteristics of patients undergoing thoracoscopic thymectomy.

Variable	ICA group (*n* = 48)	SA group (*n* = 42)	*P* value
Age, years	46.8 ± 12.3	44.9 ± 11.7	0.42
Sex (male/female)	21/27	19/23	0.88
Body mass index, kg/m^2^	23.6 ± 3.1	23.1 ± 3.4	0.51
Myasthenia gravis, *n* (%)	26 (54.2%)	25 (59.5%)	0.61
Tumor size, cm	3.2 ± 0.9	3.1 ± 1.0	0.67
Tumor laterality			0.73
Right-sided, *n* (%)	29 (60.4%)	26 (61.9%)	
Left-sided, *n* (%)	15 (31.3%)	13 (31.0%)	
Midline, *n* (%)	4 (8.3%)	3 (7.1%)	
Pathological diagnosis			0.81
Thymoma, *n* (%)	34 (70.8%)	31 (73.8%)	
Non-thymomatous lesions, *n* (%)	14 (29.2%)	11 (26.2%)	

### 3.2 Operative and perioperative outcomes

**Figure 2:**
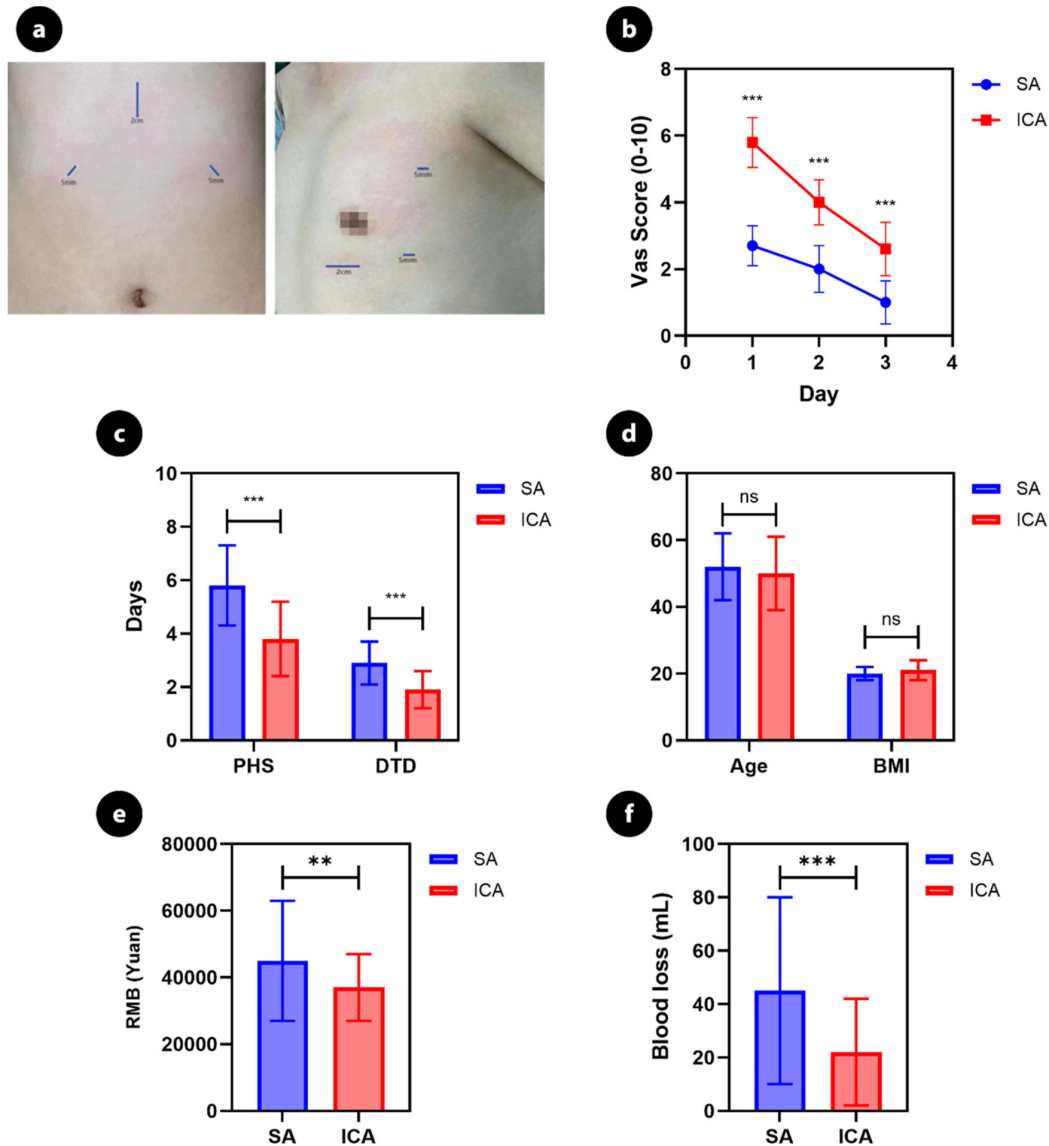
Composite comparison of surgical approaches and perioperative outcomes. Composite figure comparing the subxiphoid approach (SA) and intercostal thoracoscopic approach (ICA) for minimally invasive thymectomy. **(a)** Representative port placement; internal labels A and B depict the subxiphoid and intercostal port configurations, respectively, with port sizes indicated. **(b)** Postoperative pain trajectory assessed using the visual analog scale (VAS) over postoperative days 1–3. **(c)** Postoperative hospital stay (PHS) and drainage tube duration (DTD). **(d)** Baseline characteristics including age and body mass index (BMI). **(e)** Intraoperative blood loss. **(f)** Postoperative treatment cost (RMB). Data are presented as mean ± standard deviation. Between-group comparisons were performed using appropriate unpaired statistical tests. Statistical significance is indicated as ns (not significant), *P* < 0.01, and *P* < 0.001.

Operative and perioperative outcomes are summarized in Table 2. All procedures were completed thoracoscopically without conversion to open surgery. Mean operative time was 112 ± 28 min in the ICA group and 118 ± 31 min in the SA group (*P* = 0.34). Mean intraoperative blood loss was 78 ± 35 mL in the ICA group compared with 65 ± 30 mL in the SA group (*P* = 0.08). The duration of chest drainage was 2.6 ± 1.1 days vs. 2.4 ± 1.0 days (*P* = 0.41), and postoperative hospital stay was 5.1 ± 1.8 days vs. 4.9 ± 1.6 days (*P* = 0.56) in the ICA and SA groups, respectively. None of these perioperative parameters differed significantly between approaches. A composite comparison of operative and recovery-related outcomes is presented in Figure 2. Hospitalization costs were also comparable between groups, with no statistically significant difference observed (Figure 2e).

**Table 2:** Operative and perioperative outcomes of intercostal and subxiphoid thoracoscopic thymectomy.

Variable	ICA group (*n* = 48)	SA group (*n* = 42)	*P* value
Operative time, min	112 ± 28	118 ± 31	0.34
Intraoperative blood loss, mL	78 ± 35	65 ± 30	0.08
Conversion to open surgery, *n* (%)	0 (0)	0 (0)	—
Duration of chest drainage, days	2.6 ± 1.1	2.4 ± 1.0	0.41
Length of postoperative hospital stay, days	5.1 ± 1.8	4.9 ± 1.6	0.56

### 3.3 Postoperative pain

Postoperative pain outcomes are summarized in Table 3. VAS score at 24 h was significantly lower in the SA group (3.6 ± 1.1) compared with the ICA group (4.8 ± 1.2) (*P* < 0.001). At 48 h, VAS remained lower in the SA group (2.9 ± 0.9 vs. 3.5 ± 1.0, *P* = 0.01). The mean VAS score during the first 48 h was 3.3 ± 1.0 in the SA group and 4.2 ± 1.1 in the ICA group, corresponding to a mean difference of 0.9 points (*P* < 0.001). Rescue analgesia was required in 60.4% (29/48) of ICA patients compared with 40.5% (17/42) of SA patients (*P* = 0.04).

**Table 3:** Postoperative pain outcomes following thoracoscopic thymectomy.

Pain outcome	ICA group (*n* = 48)	SA group (*n* = 42)	*P* value
VAS score at 24 h	4.8 ± 1.2	3.6 ± 1.1	<0.001
VAS score at 48 h	3.5 ± 1.0	2.9 ± 0.9	0.01
Mean VAS during first 48 h	4.2 ± 1.1	3.3 ± 1.0	<0.001
Additional analgesic use, *n* (%)	29 (60.4%)	17 (40.5%)	0.04

### 3.4 Postoperative complications

Overall postoperative complication rates were low and comparable between groups (12.5% vs. 11.9%, *P* = 0.93) (Table 4). No severe complications (Clavien–Dindo ≥ III) or perioperative mortality occurred in either group. A detailed breakdown of complications is provided in Supplementary Table S1.

**Table 4:** Overall postoperative complications within 30 days after thoracoscopic thymectomy.

Variable	ICA group (*n* = 48)	SA group (*n* = 42)	*P* value
Any postoperative complication, *n* (%)	6 (12.5%)	5 (11.9%)	0.93
Severe complications (Clavien–Dindo ≥ III), *n* (%)	0 (0%)	0 (0%)	-

### 3.5 Short-term follow-up outcomes

Short-term follow-up outcomes are summarized in Table 5. Median follow-up duration was 12 months (IQR 9–16) in the ICA group and 13 months (IQR 10–17) in the SA group (*P* = 0.47). Among patients with myasthenia gravis, short-term improvement was observed in 69.2% (18/26) of ICA patients and 68.0% (17/25) of SA patients (*P* = 0.91). No early tumor recurrence was detected during the available follow-up period.

**Table 5:** Short-term follow-up outcomes after thoracoscopic thymectomy.

Follow-up outcome	ICA group (*n* = 48)	SA group (*n* = 42)	*P* value
Follow-up duration, months	12 (9–16)	13 (10–17)	0.47
Myasthenia gravis patients, *n*	26	25	—
MG short-term improvement, *n* (%)	18 (69.2%)	17 (68.0%)	0.91
MG no improvement, *n* (%)	8 (30.8%)	8 (32.0%)	
Early tumor recurrence, *n* (%)	0 (0)	0 (0)	—

### 3.6 Multivariable analysis

After adjustment for age, sex, tumor size, tumor laterality, and presence of myasthenia gravis, the subxiphoid approach remained independently associated with lower early postoperative pain (*β* = −0.82; 95% CI −1.24 to −0.40; *P* < 0.001). No other covariates demonstrated a statistically significant association with early postoperative pain (Table 6).

**Table 6:** Multivariable regression analysis of factors associated with early postoperative pain.

Variable	*β* coefficient	95% CI	*P* value
Surgical approach (SA vs. ICA)	−0.82	−1.24 to −0.40	<0.001
Age (per year)	0.01	−0.01 to 0.03	0.29
Sex (male vs. female)	−0.12	−0.48 to 0.24	0.51
Tumor size (per cm)	0.15	−0.05 to 0.35	0.14
Myasthenia gravis (yes vs. no)	0.09	−0.31 to 0.49	0.66
Tumor laterality (right vs. others)	0.18	−0.21 to 0.57	0.37

## 4 Discussion

The present retrospective cohort study compared perioperative and short-term outcomes between intercostal and subxiphoid thoracoscopic approaches for extended thymectomy and demonstrated that both techniques are safe and feasible, with largely comparable operative and recovery profiles. The principal finding of this analysis is that the subxiphoid approach was independently associated with lower early postoperative pain, whereas operative time, intraoperative blood loss, drainage duration, hospital stay, complication rates, and short-term neurological outcomes did not differ significantly between approaches after adjustment for baseline characteristics. These findings suggest the presence of modest procedural trade-offs rather than clear superiority of one technique over the other.

Postoperative pain represents a key determinant of early recovery following minimally invasive thoracic surgery. Even small intercostal incisions may result in intercostal nerve irritation and subsequent nociceptive stimulation, potentially affecting mobilization and patient-reported comfort (21, 22). By avoiding the intercostal spaces, the subxiphoid approach theoretically minimizes this mechanism of pain generation. Several prior reports and meta-analyses have described lower early pain scores associated with the subxiphoid technique (14–16, 23), and our findings are consistent with this body of evidence. In the present cohort, VAS scores at 24 and 48 h were significantly lower in the subxiphoid group, and rescue analgesic requirements were also reduced. Although intraoperative blood loss was numerically higher in the ICA group, the difference did not reach statistical significance (*P* = 0.08) and the absolute difference was small, suggesting limited clinical relevance.

However, the clinical magnitude of this difference warrants careful interpretation. The mean reduction in VAS during the first 48 h was 0.9 points. Published estimates of the minimal clinically important difference (MCID) for acute postoperative pain after thoracic surgery typically range from 1.0 to 1.5 points on a 10-point VAS scale. Thus, although statistically significant, the observed difference approaches the lower boundary of commonly cited MCID thresholds and may not uniformly translate into a clinically meaningful improvement for all patients. The concurrent reduction in rescue analgesia suggests a consistent but modest benefit in early postoperative comfort rather than a dramatic analgesic advantage. This nuance is important when interpreting the practical implications of our findings.

In contrast to pain outcomes, other perioperative parameters—including operative time, intraoperative blood loss, drainage duration, and length of hospital stay—were comparable between groups. Although some studies have reported prolonged operative time during the learning phase of the subxiphoid approach (16, 17), such differences were not observed in our cohort. This may reflect accumulated institutional experience and procedural standardization. Nonetheless, because the subxiphoid technique was introduced after the intercostal approach had already been established at our center, a potential learning-curve or temporal (era) effect cannot be completely excluded.

Postoperative complication rates were low and similar between groups, with no severe complications or perioperative mortality observed. These findings are consistent with prior literature supporting the safety of minimally invasive thymectomy (9, 10). Among patients with myasthenia gravis, short-term neurological improvement did not differ between approaches, suggesting comparable extent and completeness of thymic resection in the early postoperative period. However, given the median follow-up duration of approximately one year, definitive conclusions regarding long-term oncological control or sustained neurological remission cannot be drawn.

The present study contributes to the ongoing debate regarding optimal minimally invasive access for thymectomy. Existing comparative studies and meta-analyses remain heterogeneous, often limited by small sample sizes, variable patient selection, or inconsistent pain assessment methodologies (14, 16, 19, 20). By defining postoperative pain as a prespecified primary outcome and applying multivariable adjustment for clinically relevant covariates, this study provides additional clarity regarding early recovery differences between approaches. Nevertheless, the observed benefit of the subxiphoid approach appears modest in magnitude and should be interpreted within the broader context of surgical feasibility, tumor characteristics, and institutional expertise.

Several limitations merit consideration. First, the retrospective single-center design introduces potential selection bias, as surgical approach selection was influenced by tumor characteristics and surgeon preference. Although multivariable regression was performed, residual confounding cannot be fully excluded, and propensity score matching was not applied. Second, the introduction of the subxiphoid approach during the study period raises the possibility of a temporal or learning-curve effect. Third, the sample size and follow-up duration limit robust evaluation of long-term oncological safety and durable neurological outcomes. Finally, VAS-based pain assessment remains subjective, even under standardized analgesic protocols, and may be influenced by interindividual variability in pain perception. Postoperative pain was systematically recorded only within the first 48 h according to institutional protocol; longer-term pain trajectories were not available and represent a limitation of the present analysis.

Despite these limitations, the present analysis offers pragmatic clinical insight. Both intercostal and subxiphoid thoracoscopic thymectomy achieve comparable perioperative safety and short-term outcomes. The subxiphoid approach was associated with lower early postoperative pain in this cohort, but the magnitude of benefit appears moderate rather than transformative. Accordingly, surgical approach selection should be individualized, integrating tumor characteristics, surgeon expertise, institutional experience, and patient-centered priorities rather than assuming inherent superiority of one technique.

## 5 Conclusion

In this single-center retrospective cohort study, intercostal and subxiphoid thoracoscopic thymectomy demonstrated comparable perioperative safety and short-term clinical outcomes. The subxiphoid approach was associated with lower early postoperative pain in this cohort, whereas operative and recovery-related parameters did not differ significantly between techniques. Given the modest magnitude of pain reduction and the limited sample size and follow-up duration, these findings should be interpreted cautiously. Surgical approach selection should therefore be individualized, taking into account tumor characteristics, surgeon expertise, institutional experience, and patient-centered considerations rather than assuming inherent superiority of one technique.

## Data Availability

The original contributions presented in the study are included in the article/Supplementary Material, further inquiries can be directed to the corresponding author.
