# Supplementary material for: Surgical outcomes of thoracoscopic extended thymectomy via subxyphoid and left intercostal approach
Source: Front Surg. 2026 Mar 25;13:1795630. doi: 10.3389/fsurg.2026.1795630 (PMC13057270; doi:10.3389/fsurg.2026.1795630)
Supplement: Supplementary file 1 [file Table1.Docx]

## Supplementary Data

#### Table S1

**Supplementary Table S1. Detailed postoperative complications within 30 days**

| **Complication** | **ICA group (n = 48)** | **SA group (n = 42)** | **P value** |
| --- | --- | --- | --- |
| Pulmonary complications, n (%) | 3 (6.3%) | 2 (4.8%) | 0.78 |
| – Atelectasis | 2 (4.2%) | 1 (2.4%) |  |
| – Pneumonia | 1 (2.1%) | 1 (2.4%) |  |
| Cardiac complications, n (%) | 1 (2.1%) | 1 (2.4%) | 0.94 |
| Wound-related complications, n (%) | 2 (4.2%) | 2 (4.8%) | 0.89 |
| Other complications, n (%) | 1 (2.1%) | 1 (2.4%) | 0.94 |
